# Evaluation of the effectiveness of hip and knee implant models used in Catalonia: a protocol for a prospective registry-based study

**DOI:** 10.1186/s13018-019-1087-z

**Published:** 2019-02-21

**Authors:** Jorge Arias-de la Torre, Laia Domingo, Olga Martínez, Laura Muñoz, Noemí Robles, Elisa Puigdomenech, Miquel Pons-Cabrafiga, Francesc Pallisó, Xavier Mora, Mireia Espallargues

**Affiliations:** 1 Agency for Health Quality and Assessment of Catalonia (AQuAS), Barcelona, Spain; 2 CIBER Epidemiology and Public Health (CIBERESP), Madrid, Spain; 3 Institute of Biomedicine (IBIOMED), University of León, León, Spain; 4 Research Network into Health Services for Chronic Illnesses (REDISSEC), Madrid, Spain; 5 Department of Epidemiology and Evaluation, IMIM (Hospital del Mar Medical Research Institute), Barcelona, Spain; 6 eHealth Center, Universitat Oberta de Catalunya, Barcelona, Spain; 7 Orthopaedic Surgery Service, Hospital Sant Rafael, Barcelona, Spain; 8 Orthopaedic Surgery Service, University Hospital Santa María, Lleida, Spain; 9 External advisory Catalan Arthroplasty Register (RACat), Barcelona, Spain

**Keywords:** Register studies, Outlier detection, Arthroplasty, Survival analysis, Funnel plot

## Abstract

**Background:**

Monitoring results regarding the effectiveness of knee and hip arthroplasties may be useful at the clinical, economic and patient level and help reduce the number of prosthesis revisions. In Spain, and specifically in Catalonia, there is currently no systematic monitoring of the different prosthesis models available on the market. Within this context, the aim of the project presented in this protocol is to evaluate the short- and medium-term effectiveness of knee and hip models implanted in Catalonia and to identify where the results could be better or worse than expected.

**Methods:**

A prospective observational design will be drawn up based on data from a population-based arthroplasty register for hip and knee replacements that includes data from 53 of the 61 public hospitals in Catalonia. The knee and hip prosthesis models used will be identified and classified according to the type of prosthesis, fixation and, in total hip replacements, the bearing surface. For the data analysis, two methodological approaches will be used sequentially: first, an approach based on a survival analysis, followed by an approach based on standardised revision ratios and funnel plots. Following the analyses, a panel of experts will evaluate the results to identify possible sources of bias. Lastly, those models with results better or worse than expected compared to those from the comparison group will be valued, and strengths and difficulties for routine implementation of this methodology within the Catalan Arthroplasty Register will be identified.

**Discussion:**

The study presented in this protocol will allow us to identify the hip and knee prosthesis models whose results might be better or worse than expected. This information could have a potential impact at the patient, orthopaedic surgeon, healthcare manager, decision-making and industry levels, both in the short term and in the medium and long term.

## Background

Currently, knee and hip arthroplasties are one of the most frequent elective surgeries worldwide [1]. In Spain, there were 42,707 total knee arthroplasties and 41,060 total and partial hip arthroplasties performed in 2014 [2]. Various studies have confirmed that these procedures significantly improve the physical function and quality of life of the individuals operated on, are cost-effective and have good long-term results [3–5].

In the current context of continuous innovation and technological progress, the number of prosthesis models available on the market is constantly increasing [6–8]. Despite this growing trend, there are few initiatives regarding prosthetic implant repositories. At the international level, there are some repositories of the prosthetic models used to compare their effectiveness. In this sense, particular mention should be made of the UK’s and Australia’s initiatives. In the UK, aside from the National Joint Registry (NJR), supplementary bodies have also been developed, like the Orthopaedic Data Evaluation Panel (ODEP), serving as a reference point for data evaluation in monitoring primary prosthesis models for both the hip and knee, many of which are also used in our country [6, 8, 9]. Australia has the Australian Orthopaedic Association National Joint Replacement Registry (AOANJRR), which in addition to having established a systematic process to detect and confirm models with results that are worse than expected periodically publishes the results in its reports [7, 10].

Spain does not currently have a joint replacement registry covering the entire country, nor a repository of the different prosthetic models in place nationwide. Although there are regional registries, the only consolidated example is the Catalan Arthroplasty Register (RACat) [8]. The RACat is a population-based knee and hip arthroplasty registry that was launched in 2005 as a joint initiative by the Catalan Health Service (CatSalut), the Catalan Society of Orthopaedic Surgery and Traumatology (SCCOT) and the Agency for Health Quality and Assessment of Catalonia (AQuAS), the latter being responsible for the data collection, management and analysis [8, 11, 12]. The registry includes primary and revision replacements conducted at public hospitals in Catalonia, which allows for prosthesis monitoring and the calculation of effectiveness indicators like the rate of prosthesis revisions or their premature failure.

At the Catalan and Spanish level, all prosthesis models are authorised for commercialisation, but there is no systematic information to assess their post-market effectiveness and identify models with better or worse results. In general, the information available regarding efficacy and effectiveness of the different prosthetic models is provided by the manufacturers resulting from studies conducted with one or a few specific prosthetic models, or derived from studies combining different models [13–15]. As such, the monitoring of prosthesis effectiveness results based on different methodological approaches could represent the first step in identifying models with results that are potentially better or worse than expected. Also, this monitoring could allow initiating more specific analyses and actions in order to control the use of the different models, as for example, by issuing recommendations connected with the limitation of the usage of a particular model. Meanwhile, this information will be useful in promoting evidence-based clinical practice [9, 16], assisting in decision-making, fostering cost-effective practices for the National Health System and generating long-term savings by reducing the number of prosthesis revisions [17, 18].

Regarding the methodology used in the past to compare prosthesis effectiveness, various approaches to the problem have been suggested, both from a clinical perspective and from a more statistical perspective [19–27]. Two of these approaches stand out particularly for their validity and the number of studies published: techniques based on survival models and techniques using standardised ratios and funnel plots. Survival-based techniques consider the effectiveness of prostheses based on their risk of revision, taking into account potential sources of bias from a clinical approach, while also considering statistical aspects [20]. Conversely, techniques based on the use of standardised ratios and funnel plots compare ratios and take potential sources of bias into account from a fundamentally statistical approach, though they also consider clinical aspects [22, 23].

Within the context described, the main objective set for the project is to evaluate the short- and medium-term effectiveness of the models implanted and to identify those where the results could be better or worse than expected. Specifically, this study focuses on three specific objectives, which are (1) to describe the frequency of use and analyse the survival of the models used in Catalonia for knee and hip arthroplasties between 2005 and 2016 and (2) to detect specific prosthesis models with potentially better or worse than expected results. Furthermore, as a medium- and long-term objective, the proposal is (3) to identify relevant aspects for the incorporation of the methodology of this study on a routine basis within the RACat in order to identify prosthesis models that could have better or worse results.

## Methods/design

### Design and setting of the study

A prospective observational design will be drawn up based on data from the Catalan Arthroplasty Register (RACat) to describe and evaluate the functioning of the different prosthesis models used in Catalonia between 2005 and 2017. Since 2005, the RACat has systematically gathered information on knee and hip replacements implanted in publicly owned institutions in Catalonia. This information is further supplemented by data drawn from three additional sources: (1) the Central Insurance Registry (RCA) which provides information about patients’ vital records (alive, change of address, death) and enables us to track them; (2) the Minimum Basic Data Set for Hospital Discharge (MBDS-HD), which serves to complete patient data, treatment episodes and diagnoses; and (3) the RACat Prosthesis Catalogue, a compendium of information regarding the specific prosthesis models that were implanted that allows us to see their characteristics based on information provided by the manufacturers.

**Fig. 1:**
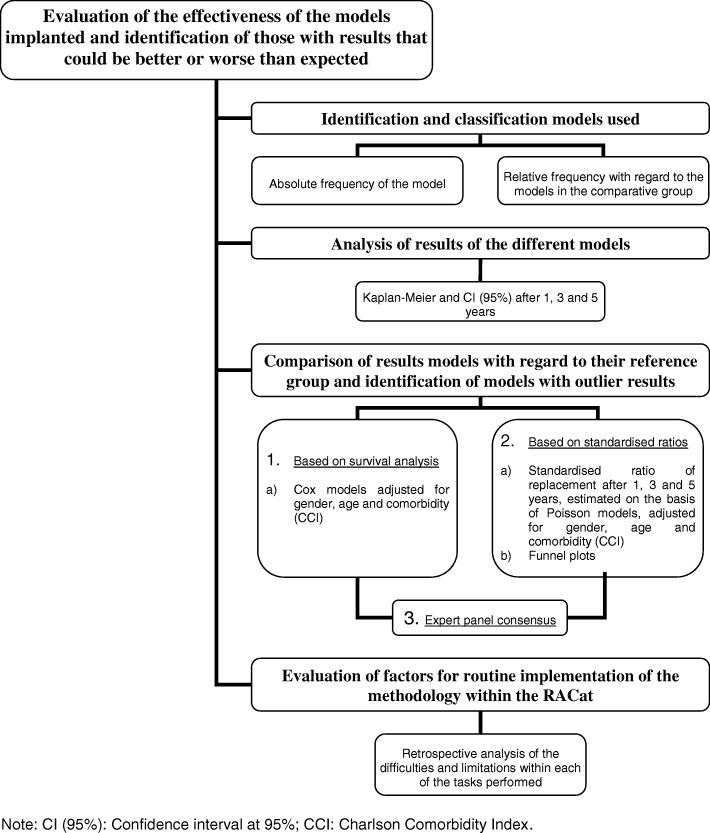
Project development phases and analysis

Firstly, the models of knee and hip prostheses used during the study period will be identified and classified according to the prosthesis group to which they belong (considering prosthesis and fixation types and, for total hip replacements, the bearing surface). Subsequently, we will analyse the results and compare the behaviour of each of the models to their reference group. Lastly, aspects and limitations related to the described process will be evaluated and taken into account for the routine implementation of this methodology within the RACat (Fig. 1).

### Study population

The study population to be considered will comprise all patients undergoing knee or hip arthroplasty operations at hospitals belonging to the SISCAT (Integral Public Health System of Catalonia) between January 2005 and December 2016. In the years following, after executing the study and verifying its utility, data analysis will continue on new cases and prosthesis models as they are added to the RACat.

The RACat currently has information available on more than 46,000 primary hip arthroplasties and 5000 revisions, and over 60,000 primary knee arthroplasties and 6500 revisions [28]. Since it was first founded, the registry has recorded more than 140 different models for total and partial hip replacement components, and around 130 different models for knee replacements, indicating the considerable variation in the models employed in Catalan public health institutions. Furthermore, according to the data from the MBDS-HD [2], irrespective of the original cause for surgery or type of prosthesis used, in 2015, publicly owned health institutions in Catalonia performed 9299 primary knee replacements and 7074 primary hip replacements, which accounted for 16.1% of all knee and hip replacements performed in Spain.

Consideration will be given to all primary prosthesis models employed throughout the period studied. The analyses will exclude cases where it is impossible to identify the operated joint and their laterality (if the operated joint is in the left or the right side), the gender of the patient undergoing the operation, their age, the presence of prior comorbidity or the specific prosthesis model. We will also exclude prosthesis models whose volume of implantation during the study period would not have allowed us to establish valid and reliable conclusions regarding the survival and possible deviation between observed and expected values. Before starting, a minimum frequency of 10 prostheses corresponding to the model implanted throughout the period studied will be established for the inclusion of a specific model within the analysis due to limitations connected with the range of the confidence intervals. This limit may vary depending on the distribution of frequencies of the specific models identified included in the RACat. Furthermore, the analyses will exclude revisions caused by infection, since the premature failure of these prostheses cannot be attributed to a poor outcome of the implant.

### Types of prostheses, fixation and bearing surface

For the grouping of the models of prosthesis and detection of results potentially better or worse than expected in comparison with the reference group, three aspects will be considered: the type of prosthesis, the fixation and, for total hip replacements, the bearing surface.

As regards the type of prosthesis, we will use the same classification system previously used by both the RACat and by studies conducted within the context of other international joint replacement registries with extensive, well-established track records [6–8], classifying prostheses according to the joint operated on, as indicated in Table 1.

**Table 1:** Types of prostheses grouped by joint operated on

Hip	Knee
Totals • Total conventional • Short stem • Surface • Double-mobility	Totals • Posterior cruciate ligament retained (CR) • Posterior cruciate ligament not retained (PS) • Constrained (TS) • Hinge • Rotational • Other types
Partials • Unipolar monoblock • Unipolar modular	Partials • Unicompartmental • Bicompartmental • Patellofemoral

Note: the types of prostheses will be grouped according to the type of fixation used, along with the bearing surface in total hip replacements

Concerning the type of fixation used for the prosthesis, the following methods will be considered: cemented (all components cemented for both hip and knee prostheses). non-cemented (none of the components cemented for either hip or knee prostheses), hybrid (only the stem cemented for hip prostheses or only the tibial component for knee prostheses) and reverse hybrid (only the acetabulum cemented in hip prostheses or only the femoral component in knee prostheses).

To analyse total hip replacements, we will also consider the bearing surface between the different components of the prosthesis, such as metal on metal, metal on polyethylene, ceramic on ceramic and ceramic on polyethylene. Moreover, in total hip prostheses comprising different components, the analysis will also take into account the combinations noted in the stem and cup models.

### Patient characteristics

We will consider the following patient characteristics: gender, age and comorbidity at the time of the operation, evaluated based on the Charlson comorbidity index (CCI) adapted for the International Classification of Diseases, 9th Edition, Clinical Modification (ICD-9-CM) [29, 30]. The CCI is a compound physical health measurement used to predict patient mortality on the basis of the comorbidity levels they reveal and is considered an objective measure of the general state of the patient’s health. The index is calculated by assigning points based on the weighted value of each of the 19 constituent conditions present, thereby serving to calculate a general morbidity index. Given the distribution of the points scores of the variable and the characteristics of the population studied, the scores obtained will be considered on a categorical basis (0 absence of comorbidity, 1–2 intermediate comorbidity, ≥ 3 high comorbidity).

### Statistical analysis

In order to achieve the objectives, the following analyses will be performed sequentially:

Firstly, the models and combinations used over the course of the period of study will be described. For each model or combination, the total frequency implanted will be calculated, along with the relative frequency according to prosthesis group and year. Furthermore, whenever the frequency of the specific prosthesis model or combination allows, the Kaplan-Meier method will be used to calculate the crude revision risk for any cause (except infection periprosthetic fracture and luxation), at 1, 3 and 5 years, with corresponding 95% confidence interval (95% CI).

Subsequently, to identify prosthesis models whose results are better or worse than expected, two different but mutually complementary methodological approaches will be employed consecutively. As the main standard to be followed, we will use the methodology proposed by de Steiger et al. [20] within the context of the Australian Registry (AOANJRR), simplified and adapted for use in the RACat. To do this, the risk of revision will be evaluated, irrespective of the reason but excluding infection, using Cox models adjusted for gender, age and CCI. Additionally, the influence of dead as a competing event with the revision of the prosthesis will be evaluated, and in the event that this fact will be verified, we will recalculate the model, considering the patient’s death as a competitive event.

Secondly, as a complementary analysis to the above methodology, a methodological approach based on standardised replacement ratios (SRR) will be used in accordance with the methodology proposed by the NJR also adapted for the specific characteristics of the RACat [27]. In this approach, a SRR between the number of replacements observed and expected will be calculated for each prosthesis model. The expected replacements will be estimated using the sum of individual replacement probabilities, after adjusting a multi-variable Poisson regression model using age, gender and comorbidity (CCI) as the adjustment variables. A SRR greater than 1 indicates that there have been more replacements than would be expected for this prosthesis model in relation to the average for equivalent models included in the RACat. In order to ensure the validity of the analyses, only those implanted models whose volume allows for subsequent comparisons (taking a priori as the minimum reference volume *n* = 10) will be included. Furthermore, we will use funnel plots to graph the SRR (*y*-axis) against the expected number of replacements (*x*-axis). Confidence intervals of 95% (95% CI) and 99% (99% CI) will be constructed, deeming that a prosthesis above or below 95% CI or 99% CI has a result that could be better or worse than expected with regard to the mean for its group. This outlier status indicates that the number of replacements performed is significantly lower or higher than the number of replacements expected with regard to the mean figure for the group (2 and 3 standard deviations, respectively, for CI 95% CI and 99% CI) [21, 23, 25]. In order to control the possible effect that the hospital might have on the results of the prostheses, a sensitivity analysis will be conducted, calculating the ratio of revision for each model with an extreme SRR with regard to the rate for other prostheses for each hospital.

Once both types of analysis have been conducted, and taking into account as the reference results those obtained on the basis of the first approach, the models obtained will be compared to determine if their results are potentially better or worse than expected. As such, the results obtained on the basis of the second methodology proposed will serve firstly as an element facilitating the visualisation of the results, and secondly as a confirmation element if the first approach reveals results that could potentially be better or worse than expected in comparison with the corresponding type.

Lastly, models identified by the first and/or second approach will be evaluated by the advisor committee of the RACat, a panel of experts in orthopaedic surgery and traumatology working with the RACat. As result of this evaluation and once a consensus about the different models will be reached, we will proceed to identify those with results potentially better or worse than expected, according to both clinical and statistical criteria, having considered possible sources of bias from both perspectives.

Once the process has been completed, and with the goal of routine implementation within the RACat, all aspects of the development of this study will be considered, in order to identify possible key points. This will entail a retrospective analysis of the difficulties and limitations encountered within each of the tasks performed so we can identify the most important aspects to take into account regarding implementation.

## Discussion

As far as we know, aside from studies that group results together by prosthesis type and clinical trials of specific prosthesis models [13–15, 31], this is the first study performed in Spain and one of the first worldwide, to determine the effectiveness of the set of knee and hip prosthesis models currently used. Additionally, this is one of the first studies dedicated to determine which of these models might have results potentially better or worse than expected in comparison with implants of the same type. This information is important given the considerable variability observed in the use of prosthesis models [28] and could prove extremely helpful in the decision-making process, both for professionals in the traumatology and orthopaedic surgery field and for administrators and planners within the National Health System. It could also be of potential interest to patients considering hip or knee replacement surgery and to prosthesis manufacturers.

Presently, there is no clear standard on the most appropriate procedure to detect models whose results could be better or worse than expected. Despite this, after reviewing the strengths and limitations of the different options previously used for this purpose [19–23, 25, 32], we have decided to use the methods described in this protocol since they offer a broad perspective from the statistical and clinical viewpoints and take into account possible sources of bias at different levels (hospital, patient, etc.). Additionally, and in order to give continuity to this study, we will attempt to routinely implement these methods within the RACat, to continuously monitor prosthesis models used in Catalonia, a process that we deem necessary given the high level of variability observed in prosthesis model usage, both within individual hospitals and among them [28].

As for the specific methods used previously, various approaches have been identified, based on different methodologies such as survival models, cumulative sum of results (CUSUM) or ratios of cases observed and expected, among others [19, 20, 24, 26]. From these approaches, at the outset, we selected the method proposed by de Steiger et al. [20], among other reasons, because this proposal was specifically designed for implementation in joint replacement registries like the RACat. Furthermore, this approach places a considerable emphasis on identifying a model with potentially better or worse than expected results in their clinical interpretation of the results, which we consider to be fundamental for an evaluation, given the possible identification of sources of bias that cannot be analysed from a statistical perspective. As a complementary analysis, and to validate the results of the survival analyses, a methodology based on the calculation of ratios between observed and expected cases derived from multi-variable regression models has been selected. From this perspective, different methods have been found to present the results. For this study, we chose funnel plots, both for practical reasons related with data visualisation and because they are the most commonly used by the studies published to date in orthopaedic surgery and traumatology [27].

Concerning the limitations of the present work, the main one is related to the results obtained from the funnel plots, as suggested by previous studies [23–25]. These results may not be as accurate as one would hope for the detection of values that deviate significantly from expectations, essentially due to limitations connected with calculating their confidence intervals. However, since this study will obtain its main results from a focus based on survival analysis, the results from the funnel plots will serve to complement this approach [26]. These graphs will be useful both as a graphical tool to help detect models with potentially worse results than their reference group and as a possible source of confirmatory information supporting the results of the survival analysis.

On the other hand, given that only prosthesis models or combinations of prosthesis models whose frequency allows us to draw valid conclusions from the analyses performed will be included, the results obtained will be limited to the most commonly used prostheses, excluding highly infrequent models. Nonetheless, a descriptive analysis of the frequency of all models included in the RACat will be performed which will therefore enable the identification of those models that given their low frequency of usage were not included. Additionally, given that the routine implementation of this methodology within the RACat is addressed, if the models remain in use in subsequent years, they could ultimately achieve a cumulative frequency of usage that would allow them to fulfil the inclusion criteria, and thus allow us to perform the proposed analyses.

With regard to the information available on joint replacement operations in the RACat, this is not complete, since the mandate to report all the prosthesis models used in public hospitals in Catalonia took effect in March 2017. However, as mentioned above, during the study period, 53 of 61 public hospitals were involved in submitting information, which accounted for more than 85% of public health procedures conducted in Catalonia [8].

In conclusion, by observing the performance of the different prosthesis models compared to the general performance observed within their reference group, our study will contribute to the current needs to identify and ascertain knee and hip prosthesis models whose results could be better or worse than expected. This identification process has a potential impact on patients, surgeons, industry and healthcare managers. Finally, we expect that this monitoring methodology could be routinely implemented to assess the different prosthesis models and promote the use of those that have positive results both in Catalonia as in the National Health System of Spain.

## Abbreviations

AOANJRR: Australian Orthopaedic Association National Joint Replacement Registry
AQuAS: Agency for Health Quality and Assessment of Catalonia
CatSalut: Catalan Health Service
CCI: Charlson comorbidity index
CI: Confidence interval
CR: Posterior cruciate ligament retained
ICD-9-CM: International Classification of Diseases, 9th Edition, Clinical Modification
MBDS-HD: Minimum Basic Data Set for Hospital Discharge
NJR: National Joint Registry
ODEP: Orthopaedic Data Evaluation Panel
PS: Posterior cruciate ligament not retained
RACat: Catalan Arthroplasty Register
RCA: Central Insurance Registry
SCCOT: Catalan Society of Orthopaedic Surgery and Traumatology
SISCAT: Integral Public Health System of Catalonia
SRR: Standardised replacement ratio
TS: Constrained
